# Taking *Conservation Physiology* forward: editorial vision of the new editor-in-chief

**DOI:** 10.1093/conphys/coae039

**Published:** 2024-07-01

**Authors:** Andrea Fuller

**Affiliations:** School of Physiology, University of the Witwatersrand, 7 York Rd, Parktown, Johannesburg, 2193, South Africa

It is a privilege to step into the role as Editor-in-Chief of the journal *Conservation Physiology*. As an Associate Editor for the past 7 years, I have particularly enjoyed seeing how *Conservation Physiology* has gone about its business, being much more than just another journal in the vast publishing landscape. Under the stewardship of the Founding Editor-in-Chief, Steven Cooke, and with the support of the editorial team, the Society for Experimental Biology (SEB), and Oxford University Press (OUP), *Conservation Physiology* has been key for the growth of the discipline (Cooke *et al.*, 2013) and the development of a thriving community of conservation physiologists. As the new Editor-in-Chief, I look to build on that position by encouraging high-quality submissions, ensuring efficient handling of manuscripts, maintaining the high standards and integrity of the journal, and promptly and respectfully communicating with authors, reviewers and the journal’s editorial and production teams.

In my capacity as an Associate Editor and conservation physiologist, I contributed to a recent article (Cooke *et al.,* 2022) in which we outlined an approach to build and strengthen a conservation physiology community that is devoted to excellence, transparency, ethics and mutual respect. We documented several commitments from the journal *Conservation Physiology* to achieve those goals, and I pledge to continue to drive those changes. In addition to those commitments, there are three main objectives that I will prioritize during my editorial tenure.

The first objective is to promote greater diversity and inclusion in conservation physiology. The journal’s editorial board has been made up mainly of experts from three countries—Australia, Canada and the USA—and those countries also are the home for the majority of authors who publish in the journal. The impact that conservation physiology can make, of course, extends to other regions. The Global South, in particular, is rich in biodiversity, but its research is poorly represented in conservation physiology and related disciplines, like ecology and conservation biology (Maas *et al.,* 2021). Those disciplines can benefit from the functional diversity among species in elucidating physiological mechanisms and developing appropriate conservation solutions. As a scientist from the Global South, I recognize the hurdles that are associated with conducting and publishing high-quality research, including the costs of Open Access. *Conservation Physiology* offers waivers for authors who are based in developing countries, and SEB members receive discounts, while OUP has a growing number of Read and Publish agreements with institutions all around the world. The journal also offers support through English language editing, where needed. I hope to promote greater diversity among the editorial team and reviewers, and increase our reach to new authors through initiatives such as special issues with a regional focus and invited contributions.

**Figure 1:**
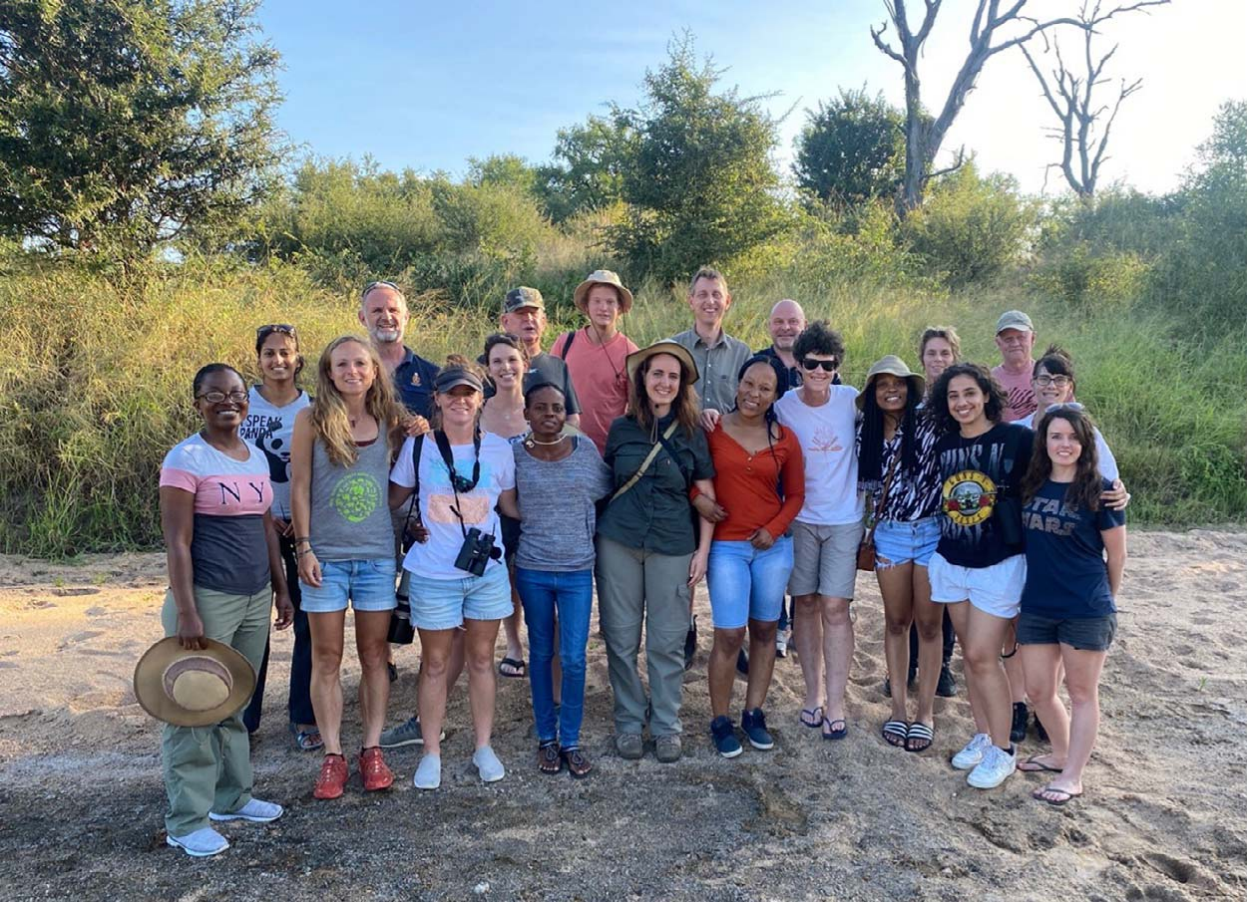
Incoming Editor-in-Chief Andrea Fuller (with sunglasses) with early-career researchers, and established scientists in conservation physiology, at a workshop in the Kruger National Park, South Africa. The workshop was supported by a grant from the Society for Experimental Biology (SEB). Conservation Physiology is partly owned by the SEB, which is supportive of early-career researchers through initiatives such as seminar presentation opportunities, training sessions, and funding for workshops and travel.

My second objective is to strengthen our platform for the support and empowerment of early-career researchers, as part of the journal’s responsibility to help nurture the next generation of conservation physiologists. I believe that *Conservation Physiology* already provides a positive experience for early-career authors through the submission and review process, and I will endeavour to maintain and strengthen those supportive aspects. The journal also supports early-career researchers through initiatives with the SEB (see Fig. 1) and encourages involvement in the editorial process, including the reviewing of articles. I plan to expand this support by providing opportunities to guest edit special issues with mentorship from the editorial team, by arranging workshops in areas of interest for young conservation physiologists, by inviting reviews from future leaders and featuring early-career researchers in our *Voices in Conservation Physiology* series.

Finally, I wish to ensure that *Conservation Physiology* is the home for impactful research in the field. The journal has published special issues, topical reviews and perspectives that have been well-received, but I would like to invite more of the leading researchers in our field to contribute syntheses to the journal. I would also like to see more submissions on underrepresented taxa in the journal, including plants, microbes and invertebrates. The impact of *Conservation Physiology* extends beyond its citation record, given that much of the research also has applied goals, and we would welcome more articles written by policy makers and practitioners. To support the impact on management and conservation practice, *Conservation Physiology* publishes a wide range of manuscript types, including methods to expand and improve the conservation physiology “toolbox”, and articles that report negative results or describe replication studies.

The academic publishing ecosystem is facing many challenges now, including impacts from easily accessible artificial intelligence platforms, and the exponential growth of publications. As a society-based journal, the primary purpose of *Conservation Physiology* is to serve conservation physiologists and to promote their science. The journal receives excellent support from Bridget O’Boyle as Assistant Editor, and Mike Page as the SEB Publications Manager, and has a reputable publisher (OUP) that is committed to high ethical standards in publishing. I look forward to working closely with this team, and to engagement with you, the members of the conservation physiology community. Please feel free to get in touch with me to discuss any initiatives to advance the discipline, ideas for manuscripts, or ways to get involved with the journal.
